# Epidermal growth factor receptor-mutant pulmonary adenocarcinoma coexisting with tuberculosis: A case report

**DOI:** 10.1097/MD.0000000000024569

**Published:** 2021-02-26

**Authors:** Ning Liu, Lingnan Zheng, Min Yu, Shuang Zhang

**Affiliations:** a Department of Thoracic Oncology; b Department of Biotherapy, State Key Laboratory of Biotherapy and Cancer Center, West China Hospital, Sichuan University, Chengdu, China.

**Keywords:** epidermal growth factor receptor tyrosine kinase inhibitors, lung cancer, pulmonary infections, tuberculosis

## Abstract

**Rationale:**

Lung cancer and pulmonary infections can have similar clinical and radiographic manifestations. Treatment for the coexistence of epidermal growth factor receptor (EGFR)-mutant pulmonary adenocarcinoma and tuberculosis remains unclear.

**Patient concerns:**

We reported a case of *EGFR-*mutant lung adenocarcinoma (mimicking pulmonary infections) that coexisted with pulmonary tuberculosis during the course of the disease.

**Diagnoses:**

The patient was initially diagnosed with pneumonia-like pulmonary adenocarcinoma with *EGFR* exon 19 deletions based on computed tomography scan, fiberoptic bronchoscopy, pathology, and genetic analysis, and then coexistence with active tuberculosis (TB) was confirmed via laboratory examinations and TB-DNA polymerase chain reaction.

**Interventions:**

Antibiotics and gefitinib were administered initially. A combination of gefitinib and anti-TB treatment was then administered when active TB was confirmed, and osimertinib was then prescribed because the disease was progressive and *EGFR T790 M* mutation was detected.

**Outcomes:**

The patient has survived with a stable disease status to date.

**Lessons:**

Exploring and ruling out differential diagnoses between pulmonary malignancies and infectious diseases is vital for treatment decisions and outcomes. The combined gefitinib-anti-TB regimen was safe, though it needed modification.

## 1 Introduction

Lung cancer is currently the most common cancer and the leading cause of cancer-related deaths worldwide.^[1]^ Pneumonia-type adenocarcinoma represents a distinct subset of lung cancer with specific pathological, radiological, and clinical features. It typically presents radiologically with varying areas of ground glass and consolidation, and the appearance is more regional than local (nodules and lumps).^[2]^

Tuberculosis (TB) is 1 of the major causes of deaths due to infectious disease and remains a global health threat.^[3]^ Reports reveal that TB infection could act as a chronic inflammatory process associated with an increased risk of lung cancer.^[4]^ A previous study indicated that pulmonary adenocarcinoma with TB lesions had a higher probability of *EGFR* mutations, especially exon 19 deletions.^[5]^ Meanwhile, in pharmacokinetics, gefitinib is metabolized by cytochrome P-450 (CYP3A4) influenced by the metabolism of rifampicin.^[6]^ Therefore, combined therapy with gefitinib and standard anti-TB treatment remains a challenge.

Accurate diagnosis and proper treatment are of great importance and directly influence the disease course and survival of patients. Usually, detailed investigations including clinical manifestations, radiographic findings, and pathological biopsy help clinicians suspect lung cancer. However, some clinical manifestations (cough, expectoration, fever, hemoptysis, weight loss, and breathlessness) and radiographic findings (solid, ground glass, or part solid and part ground glass nodules) are non-specific. Pulmonary infections and lung cancer can have similar clinical manifestations and radiographic findings.^[7]^ A wide variety of infectious diseases, including those caused by bacteria (*Mycobacterium tuberculosis)*, fungi, viruses, and parasites (rare), cause inflammatory lung lesions that are usually similar to cancer, making their differentiation difficult based on imaging.

**Figure 1:**
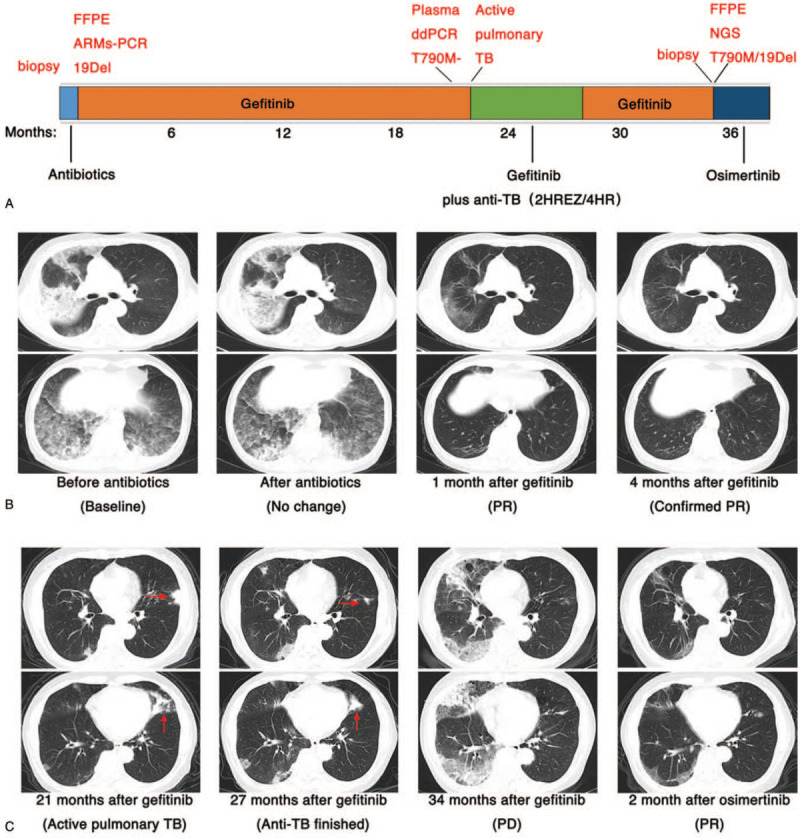
(A) Schematics showing the treatment history of the patient. (B) Chest computed tomography (CT) scan revealed the clinical response to anti-infection treatment and first-generation epidermal growth factor receptor-tyrosine kinase inhibitors (EGFR-TKIs) successively. (C) CT scan showed the clinical response to combination treatment with anti-tuberculosis (TB) agents and gefitinib, with gradual disease progress. Red arrows indicate tuberculous foci in left lung lobes. CT = computed tomography, EGFR = epidermal growth factor receptor, TKI = tyrosine kinase inhibitor.

Herein, we reported the case of a 69-year-old woman diagnosed with pneumonia-type lung adenocarcinoma with *EGFR* 19Del who had a marked response to gefitinib initially but developed active pulmonary TB. The patient received a combination of gefitinib and standard anti-TB treatment, which led to a complete resolution of tuberculosis and gradual progression of lung cancer (Fig. 1A). Our results demonstrated that the disease cannot be diagnosed too early and hence treatment cannot be initiated correctly, and biopsy samples are necessary in providing a timely and definitive diagnosis.

## 2 Case presentation

A 69-year-old Chinese woman with no history of smoking was admitted to a local hospital in January 2017 with complaints of cough, expectoration, and weight loss for 3 months. The patient was diagnosed with pneumonia, and antibiotics was prescribed. However, no signs of improvement were observed within 2 weeks of treatment. She was prompted to undergo fiberoptic bronchoscopy and was pathologically diagnosed with lung adenocarcinoma. Further genetic analyses detected *EGFR* exon 19 deletions (*EGFR* 19 del).

After admission to our hospital, a series of investigations were conducted. Laboratory examinations (routine blood tests, biochemical analyses, and fungal 1,3-β-D-glucan) were normal except for mildly raised white blood cells and elevated tumor markers (carcinoembryonic antigen and cytokeratin 19 fragment 21-1). Inflammatory markers such as C-reactive protein and procalcitonin were also elevated. Further biochemical analyses (smears for acid-fast bacilli and sputum culture for bacteria) were all negative. Chest computed tomography (CT) revealed multiple scattered nodules, ground glass opacities, patchy shadows scattered bilaterally within lung lobes, and no distant metastases. Because of the severe cough and diffuse pneumonia-like radiographic presentation, lung cancer with pneumonia was diagnosed. After 13 days of antibiotic treatment, the symptoms did not improve, and the CT scan indicated that the lung lesions were more numerous. According to the TNM staging criteria, she was diagnosed with clinical T4N0M1a, stage IV pneumonia-like lung adenocarcinoma involving different lobes in both lungs.^[2]^ She was put on gefitinib 250 mg daily on February 25, 2017. The symptoms resolved gradually after 3 days of gefitinib treatment. Chest CT showed marked regression of patchy opacities and masses in the bilateral lung lobes after 1 month of gefitinib treatment, which was evaluated as partial response. Follow-up CT after 4 months of treatment showed almost complete resolution of the lesions (Fig. 1B).

In December 2017, chest CT showed a small patchy opacity in the left upper lobe. During subsequent follow-up, the number and size of nodules in the left upper lobe gradually increased, and patchy shadows in the right lung gradually increased as well. In October 2018, liquid biopsy results indicated a negative *EGFR T790 M* mutation. In November 2018, the patient presented to our hospital again with a severe cough. Laboratory examination revealed normal values of C-reactive protein and erythrocyte sedimentation rate. Chest CT revealed multiple patchy shadows and consolidation nodules scattered bilaterally within the lung lobes. Sputum was positive for acid-fast bacilli on direct smear, and TB diagnosis was confirmed by positive results of interferon-gamma release assays and TB-DNA polymerase chain reaction. A combination of anti-TB regimen (2 months of pyrazinamide/ethambutol and 6 months of isoniazid/rifampicin, 2HRZE/4HR) and gefitinib was initiated. In the first 2 months of treatment, the cough completely resolved and the sputum smear became negative. On completion of anti-TB treatment by May 2019, patchy shadows and consolidation nodules in the left upper lobe reduced, while neoplastic lesions in the right lung increased gradually (Fig. 1C).

In December 2019, a chest CT showed exudation and consolidation in the right lung similar to the initial radiographic presentation. TB resurgence was excluded, and the patient underwent CT-guided percutaneous lung biopsy, and the pathological result was still an adenocarcinoma. Capture-based targeted sequencing was performed, and the result revealed *EGFR T790 M* and exon 19 deletions. She was switched to osimertinib (80 mg daily). After a month of treatment, CT showed partial resolution of the lesions (Fig. 1C). After 38 months of follow-up, she is still alive and maintained with second-line osimertinib treatment.

## 3 Discussion

The differential diagnoses of pulmonary infections and lung cancer have been explored for many decades and pose considerable challenges for treatment. Pathological biopsy is sometimes not suitable for patients, and there are no specific clinical manifestations or radiographic features predictive of either infection or neoplasm.^[8]^ Literature reports have shown that lung infections can easily mimic malignancies, but malignancies mimicking infections are relatively uncommon.^[9]^ Here, we reported the case of a 69-year-old female with lung adenocarcinoma whose radiological findings were consistent with pulmonary infection. Pulmonary adenocarcinoma coexisting with pulmonary infections was first considered, and antibiotic treatment led to worsening of pulmonary lesions. However, subsequent gefitinib administration provided rapid symptomatic relief and marked radiographic remission. Therefore, timely and accurate diagnosis is of great importance as it could avoid delayed treatment and disease progression.

Previous studies described the coexistence of TB and lung cancer, including TB reactivation during tyrosine kinase inhibitor (TKI) treatment in a patient with non-small cell lung cancer and in 1 with TB co-occurrence with small-cell lung cancer.^[10–11]^ In November 2018, our patient presented to our hospital with a severe cough. Chest CT revealed many patchy shadows and consolidation nodules, and the patient was suspected of cancer progression. However, positive results of TB-DNA polymerase chain reaction and smears for acid-fast bacilli confirmed the diagnosis of pulmonary TB. Current studies have proposed several explanations concerning the relationship between TB and lung cancer. First, chronic tuberculosis and fibrosis may induce genetic damage, leading to carcinogenesis of the pulmonary parenchymal tissue. Otherwise, immunocompromised status (those with human immunodeficiency virus infection, transplanted organs, and cancer) is a risk factor for pulmonary tuberculosis.^[12]^ A recent study indicated that previous pulmonary TB may be associated with more frequent *EGFR* mutations and poorer response to EGFR-TKIs in patients with pulmonary adenocarcinoma.^[13]^ Our patient was diagnosed with lung adenocarcinoma (with *EGFR* exon 19 deletions), and pulmonary TB emerged during gefitinib administration. However, the possibility of pre-existing or un identified TB cannot be completely ruled out because of the low sensitivity of smears for acid-fast bacilli. Meanwhile, the risk of TB activation should be considered in patients with solid organ malignancies, even if effective treatment is used. When the overall response to antitumor therapy is quite good, isolated sites of disease worsening or the emergence of new foci (especially in typical TB infection anatomical sites: lung upper lobes and apical lower lobes) may suggest a potential pulmonary TB infection.

Treatment of active TB in cancer patients is challenging. Rifampicin is a widely used drug for the treatment of *Mycobacterium tuberculosis* infections. However, it is a potent CYP3A4 inducer, while gefitinib is metabolized by CYP3A4.^[6]^ During coadministration of rifampicin and gefitinib, the geometric mean area under the curve for gefitinib was reduced by approximately 85% and the geometric mean maximum concentration was approximately 65%. A combination of standard anti-TB treatment and 250 mg gefitinib daily was administered to the patient. We observed good management of TB but a gradual progression of lung cancer. We supposed that a higher dose of gefitinib or a switch to another *EGFR*-TKI that is not metabolized by CYP3A4 (afatinib) could result in better clinical management.

## 4 Conclusion

We presented a case of lung adenocarcinoma mimicking infections that coexisted with pulmonary tuberculosis during the course of the disease. Clinicians should pay more attention to the differentiation between pulmonary malignancies and infectious diseases. A combination therapy for co-occurring tuberculosis and pulmonary adenocarcinoma was found to be safe, with minor modification.

## Acknowledgments

Informed consent was obtained from the patients.

## Author contributions

**Conceptualization:** Min Yu.

**Data curation:** Ning Liu, Lingnan Zheng.

**Formal analysis:** Ning Liu, Lingnan Zheng.

**Investigation:** Ning Liu, Min Yu.

**Supervision:** Shuang Zhang.

**Validation:** Min Yu, Shuang Zhang.

**Writing – original draft:** Ning Liu.

**Writing – review & editing:** Ning Liu, Shuang Zhang.
